# COX-2 is required to mediate crosstalk of ROS-dependent activation of MAPK/NF-κB signaling with pro-inflammatory response and defense-related NO enhancement during challenge of macrophage-like cell line with *Giardia duodenalis*

**DOI:** 10.1371/journal.pntd.0010402

**Published:** 2022-04-28

**Authors:** Yudan Zhao, Yongwu Yang, Min Liu, Xuening Qin, Xiran Yu, Huimin Zhao, Xiaoyun Li, Wei Li

**Affiliations:** Heilongjiang Provincial Key Laboratory of Zoonosis, College of Veterinary Medicine, Northeast Agricultural University, Harbin, China; University of Manchester, UNITED KINGDOM

## Abstract

*Giardia duodenalis*, the causative agent of giardiasis, is among the most important causes of waterborne diarrheal diseases around the world. *Giardia* infection may persist over extended periods with intestinal inflammation, although minimal. Cyclooxygenase (COX)-2 is well known as an important inducer of inflammatory response, while the role it played in noninvasive *Giardia* infection remains elusive. Here we investigated the regulatory function of COX-2 in *Giardia*-induced pro-inflammatory response and defense-related nitric oxide (NO) generation in macrophage-like cell line, and identified the potential regulators. We initially found that *Giardia* challenge induced up-regulation of IL-1β, IL-6, TNF-α, prostaglandin (PG) E2, and COX-2 in macrophages, and pretreatment of the cells with COX-2 inhibitor NS398 reduced expressions of those pro-inflammatory factors. It was also observed that COX-2 inhibition could attenuate the up-regulated NO release and inducible NO synthase (iNOS) expression induced by *Giardia*. We further confirmed that *Giardia*-induced COX-2 up-regulation was mediated by the phosphorylation of p38 and ERK1/2 MAPKs and NF-κB. In addition, inhibition of reactive oxygen species (ROS) by NAC was shown to repress *Giardia*-induced activation of MAPK/NF-κB signaling, up-regulation of COX-2 and iNOS, increased levels of PGE2 and NO release, and up-expressions of IL-1β, IL-6, and TNF-α. Collectively, in this study, we revealed a critical role of COX-2 in modulating pro-inflammatory response and defense-related NO production in *Giardia*-macrophage interactions, and this process was evident to be controlled by ROS-dependent activation of MAPK/NF-κB signaling. The results can deepen our knowledge of anti-*Giardia* inflammatory response and host defense mechanisms.

## Author summary

During microbial infections, COX-2 has been well documented as a pro-inflammatory factor, while little is known about its regulatory function and mechanism. Here we described a novel role of COX-2 in regulating *Giardia*-induced pro-inflammatory response and defense-related NO enhancement, and this process was confirmed to be associated with ROS-dependent MAPK/NF-κB activation. The findings provide invaluable insights into the mechanisms underlying anti-*Giardia* host inflammatory responses, however, more research efforts should be directed to clarify why *Giardia* infection only induces mild inflammation in the small intestine of the host, so as to fully understand the pathogenesis of giardiasis. Anyway, in combination with the anti-apoptotic role of COX-2 in enterocytes during *Giardia* infection as noted recently, we could infer that COX-2 might have clinical implications for giardiasis treatment.

## Introduction

*Giardia duodenalis* is one of the most common gastrointestinal parasites on a global scale [1], which causes an estimated 280 million cases of human infections per year [2]. The parasite also infects a broad variety of nonhuman mammals and birds and is of zoonotic concern, posing a significant threat to public health [3,4]. The life cycle of *Giardia* includes two stages: the disease-causing vegetative form, trophozoite, and the environmentally resistant and infective form, cyst [5]. *Giardia* proliferates in the small intestine and establishes an extracellular infection [6]. The majority of *Giardia* infections resolve spontaneously as a result of an effective host response, but some lead to chronic disease that typically manifests as diarrhea, abdominal pain, flatulence, weight loss, intestinal lesions, and malabsorption syndrome [2,5]. Failures in the treatment of giardiasis are becoming increasingly common and there seems to be an association between *Giardia* infection and development of irritable bowel syndrome or food allergies even after resolution [2]. The progression of giardiasis involves complex interactions between *Giardia* and the host, and current knowledge about its pathogenesis is very limited [5–7].

Infection of pathogenic microorganisms or tissue damage activates innate immune system, which recruits granulocytes including macrophages to clear microbial pathogens and injured tissue, releases inflammatory mediators such as pro-inflammatory cytokines TNF-α, IL-1β, and IL-6, reactive oxygen species (ROS), nitric oxide (NO), prostaglandins (PGs), and inflammatory enzymes including inducible NO synthase (iNOS) and cyclooxygenase (COX)-2, and arouses cellular inflammatory response to pathogens or tissue damage [8–10]. Macrophages are well known as effector cells in human and animal *Giardia* infections, which can engulf trophozoites both *in vitro* and during infection [5,11]. TNF-α and IL-6 have been reported to be essential for effective clearance of *Giardia* [12–15]. There is also a significant pyroptosis-related IL-1β and IL-18 increase induced by *Giardia* as described recently [16]. ROS contributes to a key mechanism in which phagocytic cells induce inflammation or clear pathogens [17], and it has also been recognized as an important player in *Giardia*-induced intestinal epithelial cell (IEC) apoptosis [1,18]. It is noteworthy that *Giardia*-infected individuals exhibit increased levels of NO concentration in serum [19], which might be attributed to the enhanced NO release from immune cells, possibly macrophages. Additionally, NO exhibits significant inhibition effects on *Giardia* trophozoite growth and excystation process *in vitro* [20]. There are two main COX isoforms, COX-1 and COX-2, encoded by different genes [21]. COX-1 is constitutively expressed in most tissues and appears to be responsible for the production of PGs that mediate normal physiologic functions [21]. By contrast, inducible COX-2 is expressed in most immune cells, notably macrophages, and generally considered as an important inducer of inflammation through converting arachidonic acid into pro-inflammatory PGs, majorly PGE2, and inducing generation of other pro-inflammatory chemokines and cytokines [21]. In spite of the common inflammation-inducing function of COX-2, it remains to be investigated whether COX-2 can act as an effective modulator affecting the levels of pro-inflammatory cytokines and NO that are necessary for *Giardia* clearance, and if this occurs, the potential upstream controllers for COX-2 regulation are also worthy of being explored, such as MAPK, NF-κB, and ROS signaling.

MAPK pathways are critical for regulating expressions of COX-2, pro-inflammatory cytokines, and iNOS, and play a role in initiating and sustaining inflammatory response and providing defense against certain pathogens [22,23]. The inflammatory response mediated by NF-κB signaling is essential for host defense against pathogens [24]. During parasitic infections, NF-κB activation was known to influence the relationship between host and pathogens through regulating innate immunity and inflammation [25]. NF-κB activity was proved to be involved in the regulation of COX-2 and iNOS expression [26,27]. However, in the context of *Giardia* infection, nothing is known about the involvement of MAPK/NF-κB signaling in regulation of COX-2 expression and potential COX-2-mediated pro-inflammatory cytokine and NO production in macrophages. In addition to the regular role of ROS as described earlier, it is also able to operate as both a signaling molecule and a mediator of inflammation [17]. It has been reported that ROS-dependent activation of MAPK/NF-κB signaling provides contributions to *Propionibacterium acnes*-induced COX-2/PGE2 and iNOS/NO in macrophages [28]. Thus, it is also worth studying the correlation of ROS signaling with MAPK/NF-κB activation, as well as its influence on the potential COX-2-mediated anti-*Giardia* host defense responses in macrophages.

To date, the regulators in inflammatory response of macrophages to *Giardia* infection are still largely unknown. The objective of this study is to analyze the potential function of COX-2 in mediating *Giardia duodenalis*-induced pro-inflammatory response and defense-related NO production in macrophage-like cell line, and to determine if this process is regulated by ROS-dependent MAPK/NF-κB signaling.

## Methods

### Cell culture

The macrophage J774A.1 and RAW264.7 cell lines [29] used to interact with *Giardia* trophozoites in this study were purchased from the Cell Bank of the Chinese Academy of Sciences (Shanghai, China). J774A.1 and RAW264.7 cells were cultured in high-glucose DMEM (Hyclone, Logan, USA) containing 10% heat-inactivated FBS (Cellmax, Beijing, China) and maintained in a 37°C humidified incubator with 5% CO_2_. Cell lines were passaged using 0.25% trypsin (Beyotime, Shanghai, China) on reaching nearly 80% confluency. Cells from passages 3–6 were used.

### Parasite culture

*G*. *duodenalis* WB strain genotyped as assemblage A was used in this study (ATCC 30957, Manassas, USA). Trophozoites were axenically cultivated at 37°C in 15 mL conical tubes in modified TYI-S-33 culture medium containing 10% FBS and 0.1% bovine bile supplemented with 0.1% gentamycin and 1% penicillin/streptomycin [30]. Parasite cultures were harvested by chilling on ice for 15 min. Detached trophozoites were centrifuged, washed with PBS, resuspended in cell culture medium, counted using a hematocytometer, and used to interact with macrophages at a ratio of 10 parasites/cell. In time-course experiments, trophozoites were added at different time points and cells harvested together.

### qPCR analysis

Cells were seeded in 12-well plates (5 × 10^5^ cells/well), incubated for 12 h, exposed to *Giardia* for the indicated time periods, and washed with ice-cold PBS to remove parasites. Total RNA was isolated using Trizol reagent (Invitrogen, Carlsbad, USA). cDNA was synthesized from the total RNA (~1 g) by reverse transcription using a HiScript II 1st Strand cDNA Synthesis Kit (Vazyme, Nanjing, China). Primers used for qPCR (S1 Table) were designed using NCBI primer BLAST tool and synthesized by SangonBiotech (Shanghai, China). qPCR was performed using a SYBR-Green PCR Master Mix Kit (Vazyme, Nanjing, China) on a LC480 Lightcycler system (Roche, Indianapolis, USA). The expression of target genes relative to the housekeeping gene, β-actin, was analyzed according to the 2^-ΔΔCt^ method.

### Western blot analysis

Cells were seeded in 6-well plates (1 × 10^6^ cells/well), incubated for 12 h, exposed to *Giardia* for the indicated time periods, and washed with ice-cold PBS to remove parasites. Total cellular proteins were extracted from each group of cells using RIPA lysis buffer (Beyotime, Shanghai, China) containing protease inhibitor (1% PMSF; Beyotime, Shanghai, China). Protein concentration was determined by an enhanced BCA Protein Assay Kit (Beyotime, Shanghai, China). Protein expression levels were measured by western blot analysis. In brief, proteins were separated by 12% SDS-PAGE and electro-transferred to PVDF membranes. Membranes were blocked with 5% skim milk in PBST for 2 h at room temperature (RT), followed by overnight exposure to primary antibodies against IL-1β (1:500 dilution in PBST), IL-6 (1:500), TNF-α (1:500), β-actin (1:1000), COX-2 (1:500), iNOS (1:500), p38 (1:1000), p-p38 (1:1000), ERK1/2 (1:1000), p-ERK1/2 (1:1000), NF-κB (1:1000), and p-NF-κB (1:1000) at 4°C. Primary antibodies were acquired from two main commercial sources (ABclonal, Wuhan, China; Bioss, Beijing, China). Membranes were washed three times in PBST and probed with HRP-conjugated secondary antibody (1,5000; ABMART, Shanghai, China) for 1 h at RT. Images were obtained with a GeneGnome XRQ chemiluminescence imaging system (Syngene, Cambridge, UK) and the intensity of the detected bands quantified with the NIH Image J software.

### PGE2 measurement

Cells were cultured in 6-well plates (1 × 10^6^ cells/well), incubated for 12 h, and exposed to *Giardia* for the indicated time periods. PGE2 concentration in the culture supernatants was detected by a mouse PGE2 ELISA Kit (CUSABIO, Wuhan, China) according to the manufacturer’s instructions. The optical density of each well was measured using a microplate reader at 450 nm wavelength within 10 min.

### Protein inhibition

COX-2 inhibitor NS398 (50 μM, final concentration), p38 inhibitor SB202190 (10 μM), ERK1/2 inhibitor SCH772984 (10 μM), and NF-κB inhibitor JSH-23 (50 μM) (Selleckchem, Houston, USA), as well as ROS inhibitor NAC (10 μM; APEXBIO, Houston, USA) were used to inhibit target proteins in this study. All inhibitors were applied 1 h prior to *Giardia* exposure.

### NO measurement

Cells were seeded in 96-well plates (1 × 10^4^ cells/well), cultured for 12 h, and exposed to *Giardia* for the indicated time periods. NO production represented by nitrite concentration in supernatants of cultured macrophages was assayed with Griess reaction using a NO Assay Kit (Beyotime, Shanghai, China). The absorbance was measured at the wavelength of 540 nm.

### ROS measurement

Cells were seeded in 24-well plates at a density of 1 × 10^5^ per well. After 12 h incubation, cells were exposed to *Giardia* for the indicated time periods and washed with ice-cold PBS to remove parasites. The intracellular ROS levels were measured using an oxidation-sensitive fluorescent probe DCFH-DA Kit, and Rosup was included as a positive control (Beyotime, Shanghai, China). The DCF fluorescence intensity in cells was measured using a Lionheart FX Automated Microscope (BioTek, Winooski, USA). Intracellular ROS levels were also detected by flow cytometry on a BD FACS Canto II (BD Biosciences, San Jose, USA). Data were analyzed using the BD FACSDiva software program (BD Pharmingen, San Diego, USA), and then processed using the Flowjo software (Tree Star, Ashland, USA).

### Immunofluorescence assays

Cells in 24-well plates (1 × 10^5^ cells/well) were challenged with parasites for the indicated time periods, washed with ice-cold PBS to remove parasites, fixed with 4% paraformaldehyde in PBS for 30 min at RT, and permeabilized with 0.25% Triton-X 100 in PBS for 10 min at RT. Nonspecific binding sites were blocked by incubation in 1% BSA in PBS for 1 h at RT. Cells were incubated with anti-NF-κB antibody (dilution 1:200) with 1% BSA in PBST overnight at 4°C, and then FITC-AffiniPure Goat Anti-Rabbit IgG (H + L) (dilution 1:200; Jackson, West Grove, USA) at 37°C for 1 h in the dark. Cell nucleus was stained by DAPI (2 μg/mL; Alphabio, Tianjin, China). Fluorescence images were captured and analyzed using a Lionheart FX Automated Microscope.

### Statistical analysis

Statistical analyses were performed using the GraphPad Prism 7.0 program. Data from triplicate wells from a representative of at least three independent experiments are presented as means ± standard deviation (SD). The statistical significance of the differences was evaluated by the use of Student’s t-test in comparison of two groups or one-way ANOVA in comparison of three or more groups. *p*-values less than 0.05 were considered to be statistically significant (* *p* < 0.05, ** *p* < 0.01).

## Results

### *Giardia* induced pro-inflammatory cytokine expression

**Fig 1:**
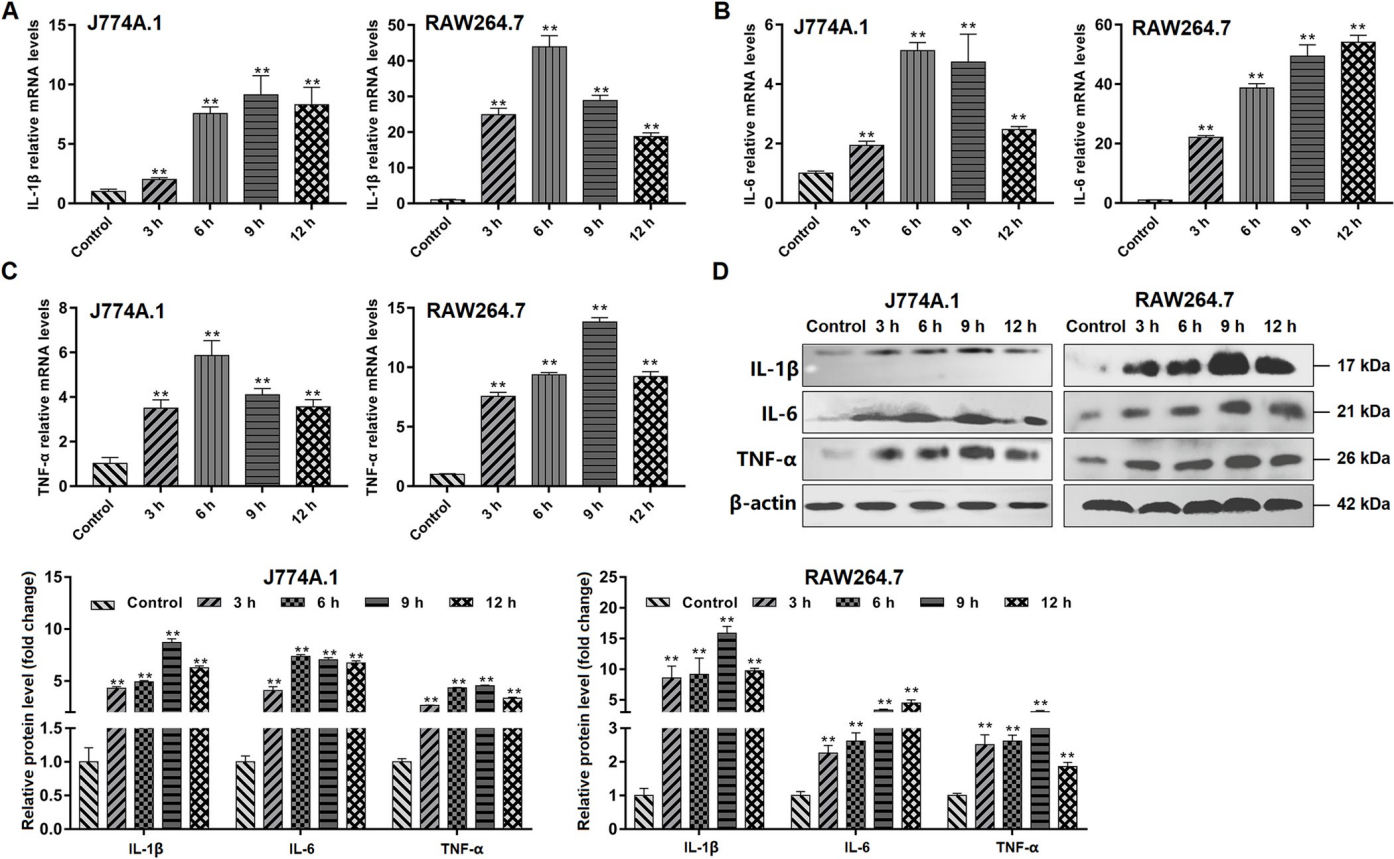
*Giardia* induced pro-inflammatory cytokine expression. (A-D) Upon *Giardia* trophozoite exposure for the indicated time periods, the mRNA and protein levels of IL-1β, IL-6, and TNF-α in J774A.1 and RAW264.7 cells were measured by qPCR, western blot, and gray value analyses. All results were normalized against the level of β-actin. Data from triplicate wells from a representative of at least three independent experiments are presented as means ± SD. ** *p* < 0.01.

We performed qPCR and western blot analyses to assess the effects of *Giardia* challenge on the induction of pro-inflammatory cytokine expression in macrophages. The levels of mRNA transcription and protein expression of the pro-inflammatory cytokines IL-1β, IL-6, and TNF-α were markedly increased in all *Giardia*-exposed groups compared with the controls (*p* < 0.01, Fig 1). The mRNA levels of these cytokines in RAW264.7 cells increased more obviously than those in J774A.1 cells and peaked at different time points between the two cell types (Fig 1A to 1C). The change trends of protein levels of IL-1β and TNF-α at different time points were similar between J774A.1 and RAW264.7, while this is not the case for IL-6 (Fig 1D). In addition, we often observed large fold differences in mRNA levels with much more minor effects on protein levels (Fig 1). Taken together, the data implied that *Giardia* exposure exerted a pro-inflammatory effect on different macrophage-like cell lines.

### COX-2 regulated pro-inflammatory cytokine expression

**Fig 2:**
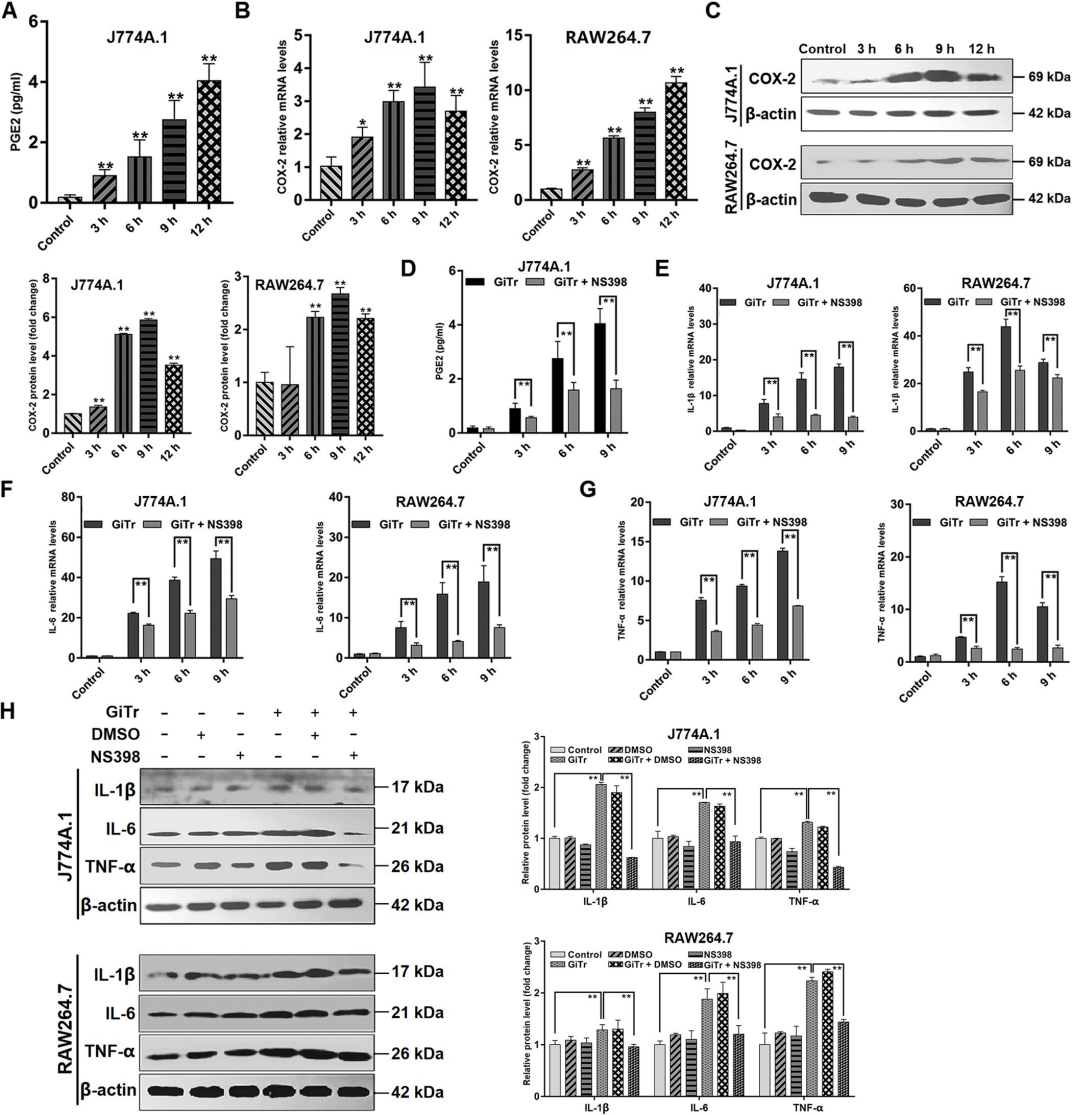
COX-2 regulated pro-inflammatory cytokine expression. Unless otherwise specified, J774A.1 and RAW264.7 cells were challenged with *Giardia* trophozoites for the indicated time periods. (A) *Giardia* exposure induced PGE2 level changes as examined by enzyme immunoassay. (B,C) Upon *Giardia* challenge, the mRNA and protein levels of COX-2 were measured by qPCR, western blot, and gray value analyses. (D) COX-2 inhibition by its inhibitor NS398 affected *Giardia*-induced PGE2 enhancement as assessed by enzyme immunoassay. (E-G) COX-2 inhibition affected *Giardia*-induced changes of IL-1β, IL-6, and TNF-α mRNA levels. (H) At 9 h after *Giardia* challenge, COX-2 inhibition affected the up-regulated protein levels of IL-1β, IL-6, and TNF-α as assessed by western blot and gray value analyses. The results of qPCR and western blot analyses were normalized against the level of β-actin. Data from triplicate wells from a representative of at least three independent experiments are presented as means ± SD. * *p* < 0.05, ** *p* < 0.01. “GiTr” reads *Giardia* trophozoite.

COX-2 has been studied generally as an inflammatory inducer [31], the role it played in regulating inflammatory responses to microbial infections is still largely not understood. In the present study, we initially assessed if *Giardia* challenge could induce PGE2 production and COX-2 expression. Released PGE2 was detected by enzyme immunoassay, and COX-2 expression levels were analyzed by qPCR and western blotting. The results exhibited that *Giardia* triggered a significant time-dependent increase in PGE2 production in J774A.1 cells (*p* < 0.01, Fig 2A). Significant up-regulation of COX-2 expression was observed at both mRNA and protein levels in J774A.1 and RAW264.7 cells exposed to *Giardia* as early as 3 h after exposure, and in both cell types, maximal expression was observed almost at 9 h post-exposure (*p* < 0.01, Fig 2B and 2C). We further evaluated the role of COX-2 in regulating macrophage pro-inflammatory response during *Giardia* infection by the use of COX-2 inhibitor NS398. As expected, COX-2 inhibition significantly attenuated the up-regulated production of PGE2 in J774A.1 cells (*p* < 0.01, Fig 2D) and the elevated mRNA and protein expression levels of IL-1β, IL-6, and TNF-α in both cell types (*p* < 0.01, Fig 2E–2H) induced by *Giardia*. The data implied that COX-2 could be an important inflammatory regulator in macrophages during *Giardia* infection.

### COX-2 regulated NO production and iNOS expression

**Fig 3:**
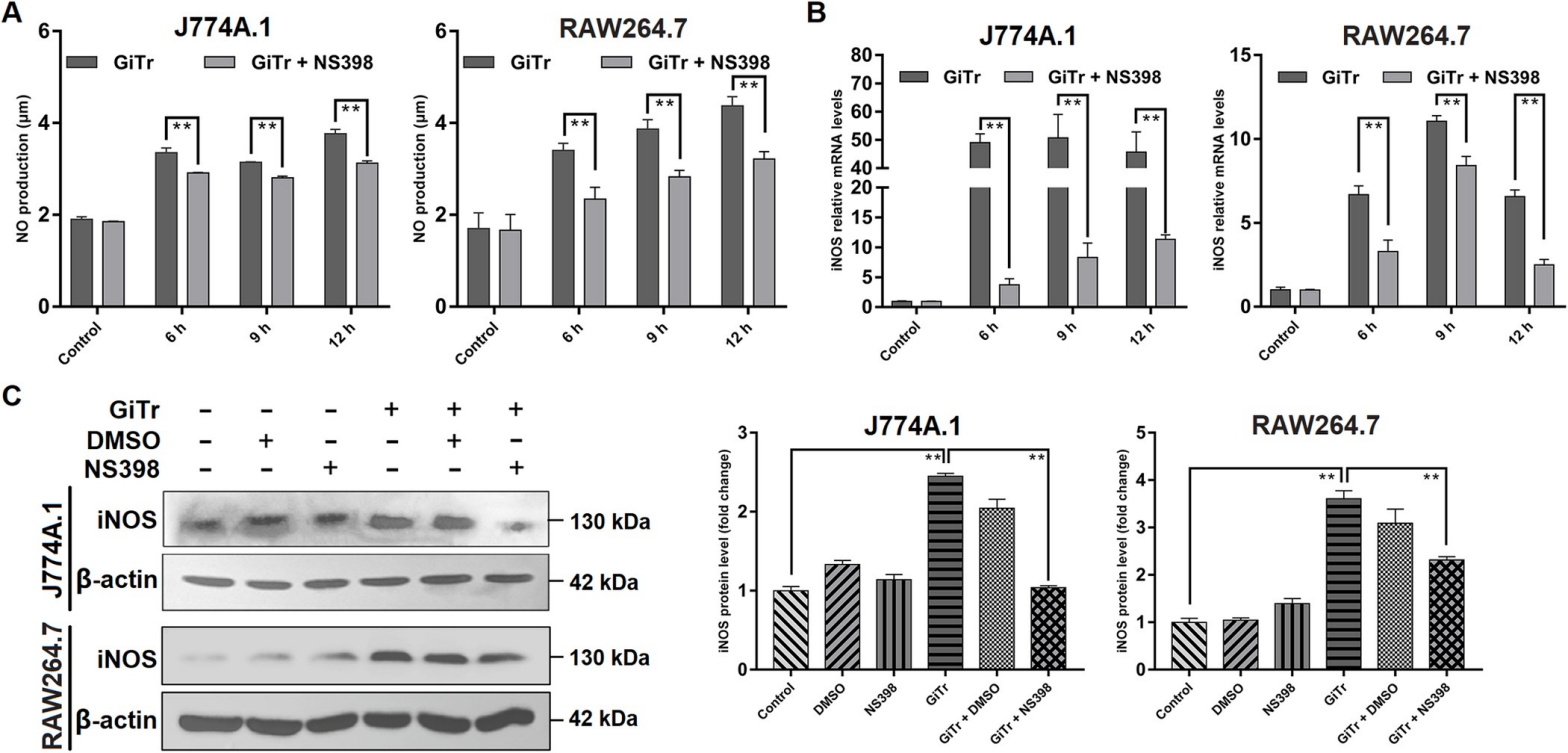
COX-2 regulated NO production and iNOS expression. Unless otherwise specified, J774A.1 and RAW264.7 cells were exposed to *Giardia* trophozoites for the indicated time periods. (A) COX-2 inhibition affected *Giardia*-induced increase of NO release as measured with Griess reagent method. (B) COX-2 inhibition affected the up-regulated iNOS mRNA level induced by *Giardia*. (C) At 9 h *Giardia* post-exposure, COX-2 inhibition affected the up-regulated protein level of iNOS as assessed by western blot and gray value analyses. The results of qPCR and western blot analyses were normalized against the level of β-actin. Data from triplicate wells from a representative of at least three independent experiments are presented as means ± SD. ** *p* < 0.01. *Giardia* trophozoite is abbreviated as “GiTr”.

NO expression by iNOS is a vital host defense mechanism against *Giardia* infection [5], thus it is quite interesting to identify its upstream regulator. In this study, NO release was measured by means of the Griess reaction, which was significantly increased following exposure of J774A.1 and RAW264.7 cells to *Giardia* within hours, whereas COX-2 inhibition by NS398 resulted in a remarkable decrease in NO release (*p* < 0.01, Fig 3A). In addition, inhibition of COX-2 activity by NS398 dramatically weakened the increased mRNA and protein levels of iNOS in J774A.1 and RAW264.7 cells exposed to *Giardia* (*p* < 0.01, Fig 3B and 3C), supporting the role of COX-2 as a positive NO regulator.

### MAPK/NF-κB signaling regulated COX-2 expression

**Fig 4:**
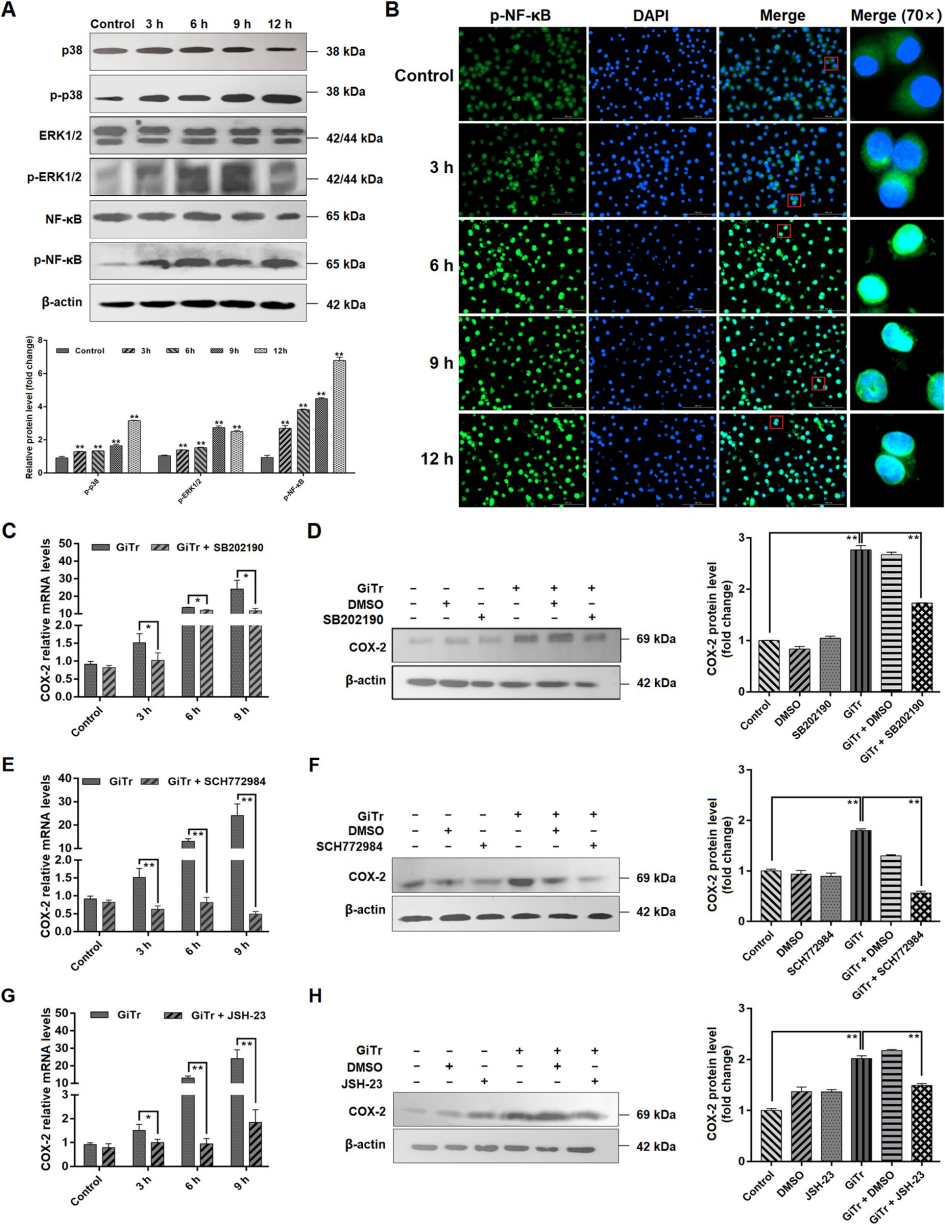
MAPK/NF-κB signaling regulated COX-2 expression. Unless otherwise specified, J774A.1 cells were challenged with *Giardia* trophozoites for the indicated time periods. (A) *Giardia* challenge affected the phosphorylation levels of p38, ERK, and NF-κB as assessed by western blot and gray value analyses. (B) *Giardia* exposure affected NF-κB nuclear translocation as assayed by indirect immunofluorescence staining (scale bar = 100 μm). (C-H) p38 inhibition by SB202190, ERK inhibition by SCH772984, and NF-κB inhibition by JSH-23 affected *Giardia*-induced up-regulation of COX-2. (C,E,G) p38, ERK, or NF-κB inhibition affected *Giardia*-induced up-regulated mRNA level of COX-2. (D,F,H) At 9 h after *Giardia* challenge, p38, ERK, or NF-κB inhibition influenced the up-regulated protein level of COX-2 as assessed by western blot and gray value analyses. The results of qPCR and western blot analyses were normalized against the level of β-actin. Data from triplicate wells from a representative of at least three independent experiments are presented as means ± SD. * *p* < 0.05, ** *p* < 0.01. “GiTr” reads *Giardia* trophozoite.

Activation of MAPK/NF-κB signaling is well known to be essential for host defense against microbial pathogens by inducing the production of pro-inflammatory cytokines [32,33], while whether it can act as a COX-2 controller during *Giardia* infection is still not clear. In this study, we initially observed the activation of MAPK (p38 and ERK1/2) and NF-κB signaling in J774A.1 cells interacting with *Giardia*. The phosphorylation of p38, ERK1/2, and NF-κB was continuously increased following exposure (*p* < 0.01, Fig 4A). As shown in Fig 4B, *Giardia* challenge also promoted nuclear translocation of NF-κB. In addition, inhibitions of p38 with its inhibitor SB202190, ERK1/2 with its inhibitor SCH772984, and NF-κB with its inhibitor JSH-23 all significantly repressed *Giardia*-induced up-regulation of COX-2 expression at both mRNA and protein levels (*p* < 0.05, Fig 4C to 4H), while p38 inhibition appeared to display a minimal effect compared to ERK/NF-kB inhibition. The results implicated that MAPK/NF-κB signaling involved regulation of COX-2 in the present context.

### ROS influenced the activation of MAPK/NF-κB signaling

**Fig 5:**
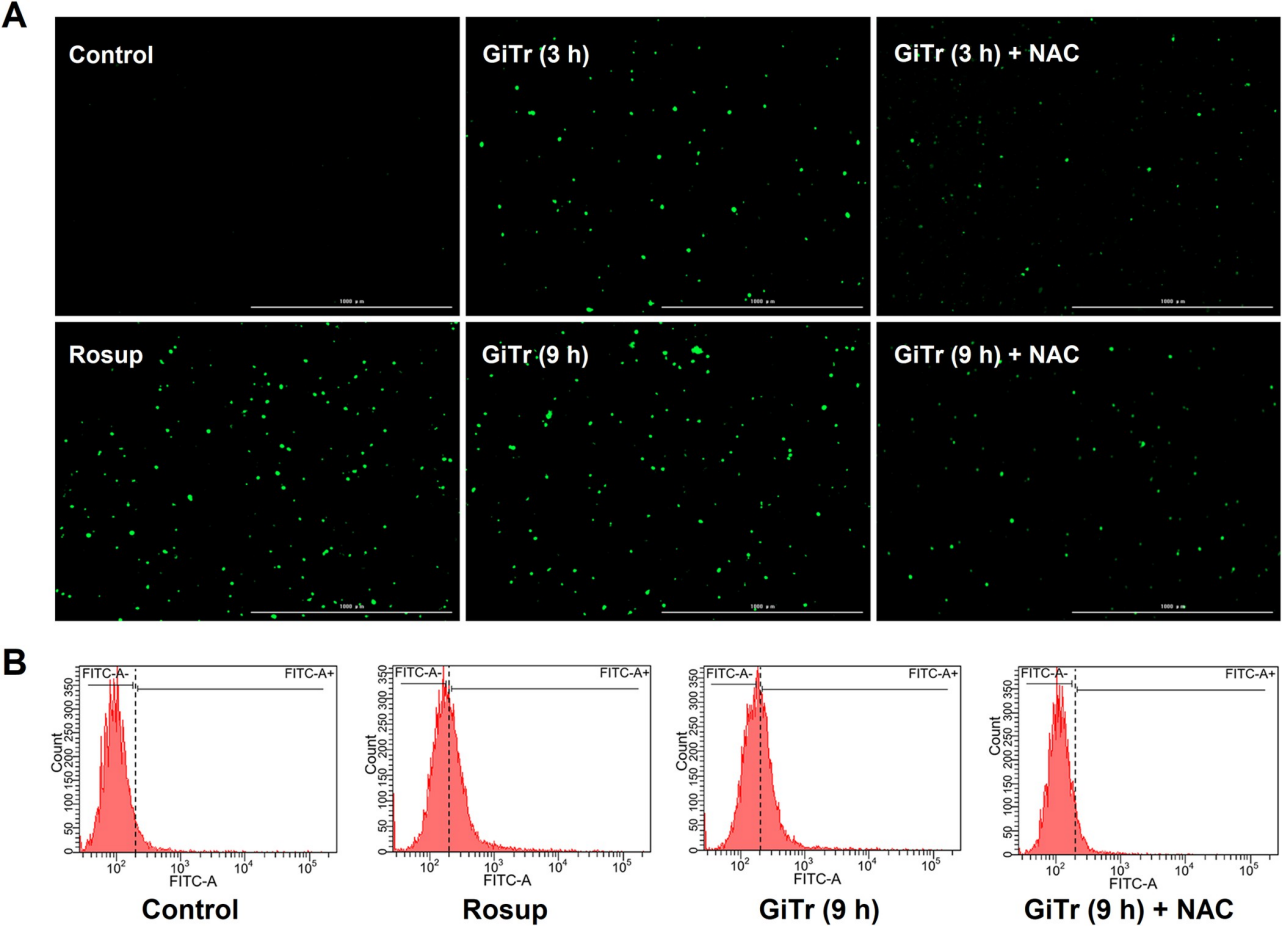
*Giardia* triggered ROS accumulation in J774A.1 cells. Prior to exposure to *Giardia* for the indicated time periods, cells were incubated with or without ROS inhibitor NAC for 1 h. Mock groups were included. (A) ROS level was examined by fluorescence microscopy (scale bar = 1000 m). (B) ROS level was examined by flow cytometry. Data from triplicate wells from a representative of at least three independent experiments are presented. *Giardia* trophozoite is abbreviated as “GiTr”.

**Fig 6:**
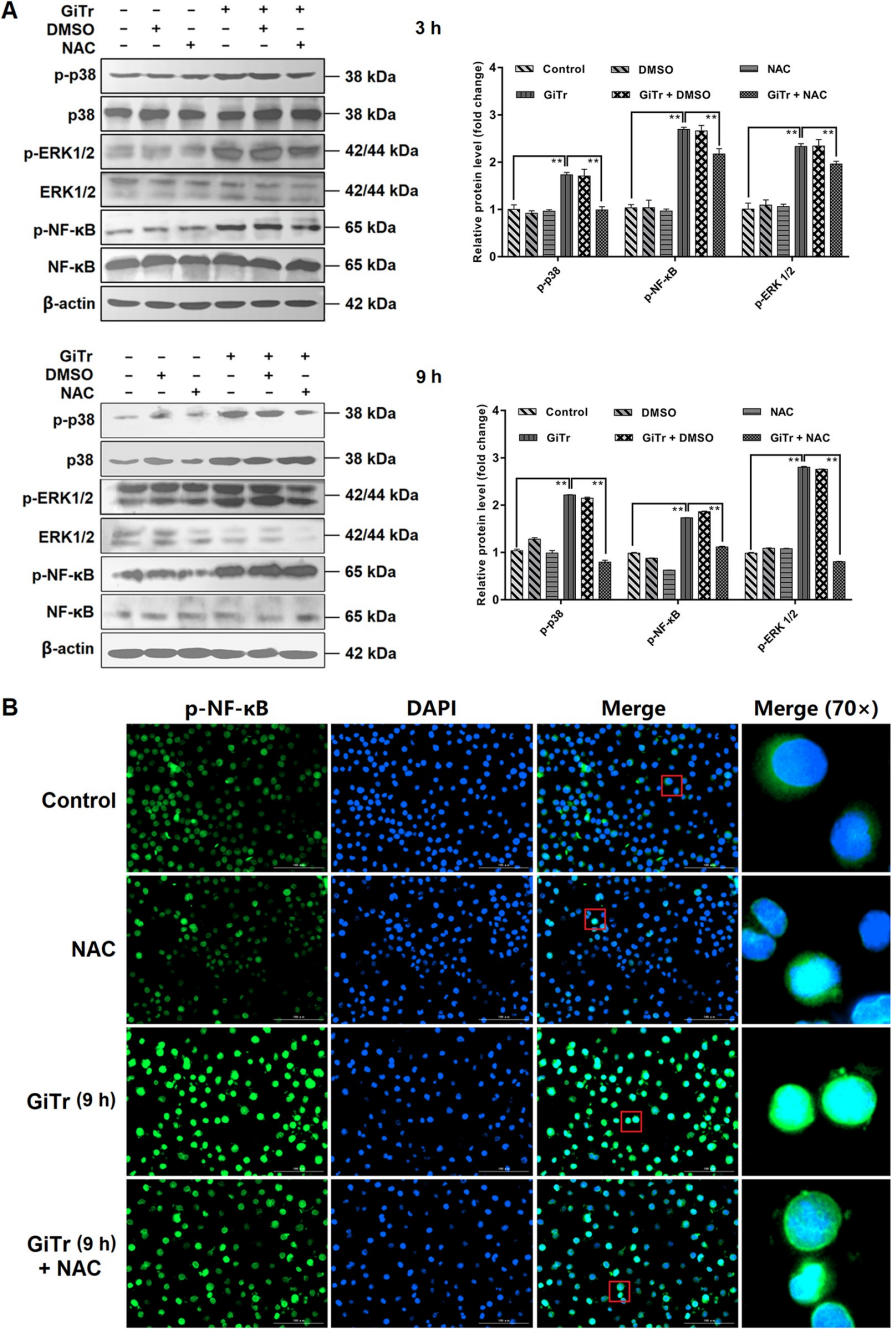
ROS influenced the activation of MAPK/NF-κB signaling. Prior to exposure to *Giardia* for the indicated time periods, J774A.1 cells were incubated with or without ROS inhibitor NAC for 1 h. Mock groups were included. (A) ROS inhibition affected *Giardia*-induced p38, ERK, or NF-κB activation as assessed by western blot and gray value analyses. (B) ROS inhibition affected *Giardia*-induced NF-κB nuclear translocation as examined by indirect immunofluorescence staining (scale bar = 100 μm). The results of western blot analysis were normalized against the level of β-actin. Data from triplicate wells from a representative of at least three independent experiments are presented as means ± SD. ** *p* < 0.01. *Giardia* trophozoite is abbreviated as “GiTr”.

As measured using DCFH-DA method and observed in Fig 5A and 5B, ROS levels were obviously increased in J774A.1 cells as early as 3 h after *Giardia* exposure, and NAC (ROS inhibitor) pretreatment could inhibit *Giardia*-induced ROS accumulation. In addition, ROS inhibition by NAC markedly repressed *Giardia*-induced MAPK/NF-κB activation (*p* < 0.01, Fig 6A), with more significant effects seen on p38 than on ERK1/2 or NF-Κb at 3 h after exposure as well as on p38 or ERK1/2 than on NF-Κb at 9 h after exposure. It was also observed that ROS inhibition could suppress *Giardia*-induced NF-κB nuclear translocation (Fig 6B). The results suggested that ROS played an imperative role in regulating MAPK/NF-κB signaling in *Giardia*-macrophage interactions.

### ROS mediated regulation of COX-2, PGE2, iNOS, NO, IL-1β, IL-6, and TNF-α

**Fig 7:**
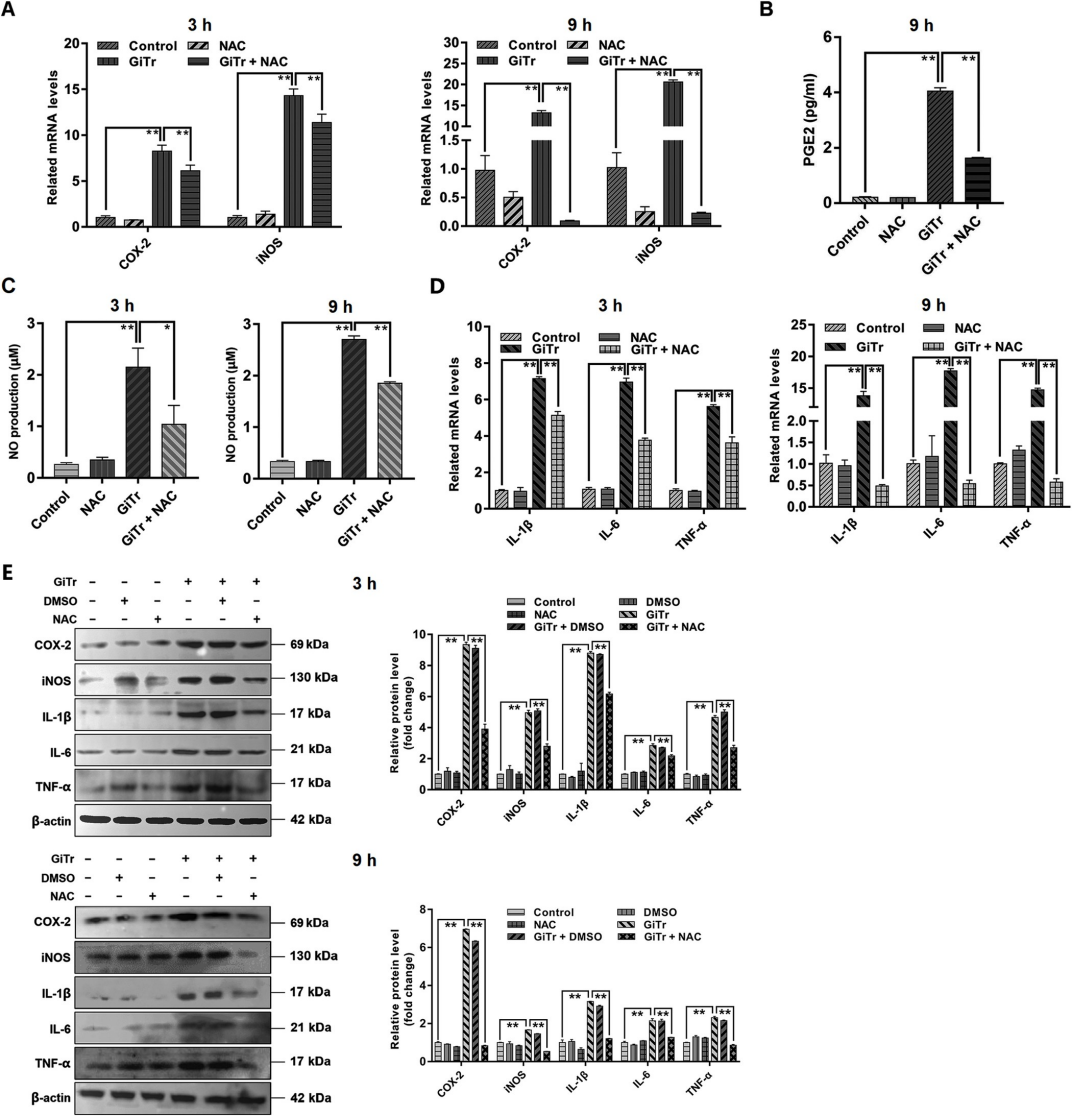
ROS mediated regulation of COX-2, PGE2, iNOS, NO, IL-1β, IL-6, and TNF-α. Prior to exposure to *Giardia* for the indicated time periods, J774A.1 cells were incubated with or without ROS inhibitor NAC for 1 h. Mock groups were included. (A) ROS inhibition affected the up-regulated mRNA levels of COX-2 and iNOS induced by *Giardia*. (B) ROS inhibition affected *Giardia*-induced PGE2 enhancement as examined by enzyme immunoassay. (C) ROS inhibition affected the increased NO level induced by *Giardia*. (D) ROS inhibition influenced the up-regulated mRNA levels of IL-1β, IL-6, and TNF-α induced by *Giardia*. (E) ROS inhibition affected the up-regulated protein levels of COX-2, iNOS, IL-1β, IL-6, and TNF-α induced by *Giardia* as assessed by western blot and gray value analyses. The results of qPCR and western blot analyses were normalized against the level of β-actin. Data from triplicate wells from a representative of at least three independent experiments are presented as means ± SD. * *p* < 0.05, ** *p* < 0.01. *Giardia* trophozoite is abbreviated as “GiTr”.

In addition to the impact of ROS on MAPK/NF-κB activation just assessed, we also examined the potential function of ROS in modulating macrophage inflammatory mediators during *Giardia* infection. It was shown that ROS inhibition by NAC remarkably suppressed the up-regulated COX-2 and iNOS expressions and PGE2 and NO release in *Giardia*-exposed J774A.1 cells (*p* < 0.05, Fig 7A–7C and 7E). *Giardia*-induced up-expression of pro-inflammatory cytokines IL-1β, IL-6, and TNF-α was found to be strikingly inhibited by NAC application (*p* < 0.01, Fig 7D and 7E). Interestingly, when NAC was applied, a more significant decrease in the mRNA and protein expressions of COX-2, iNOS, IL-1β, IL-6, and TNF-α was seen at 9 h after *Giardia* exposure than at 3 h after exposure (Fig 7A, 7D, and 7E). Considering the earlier findings, it can be inferred that ROS functioned vitally in *Giardia*-induced macrophage inflammation.

## Discussion

**Fig 8:**
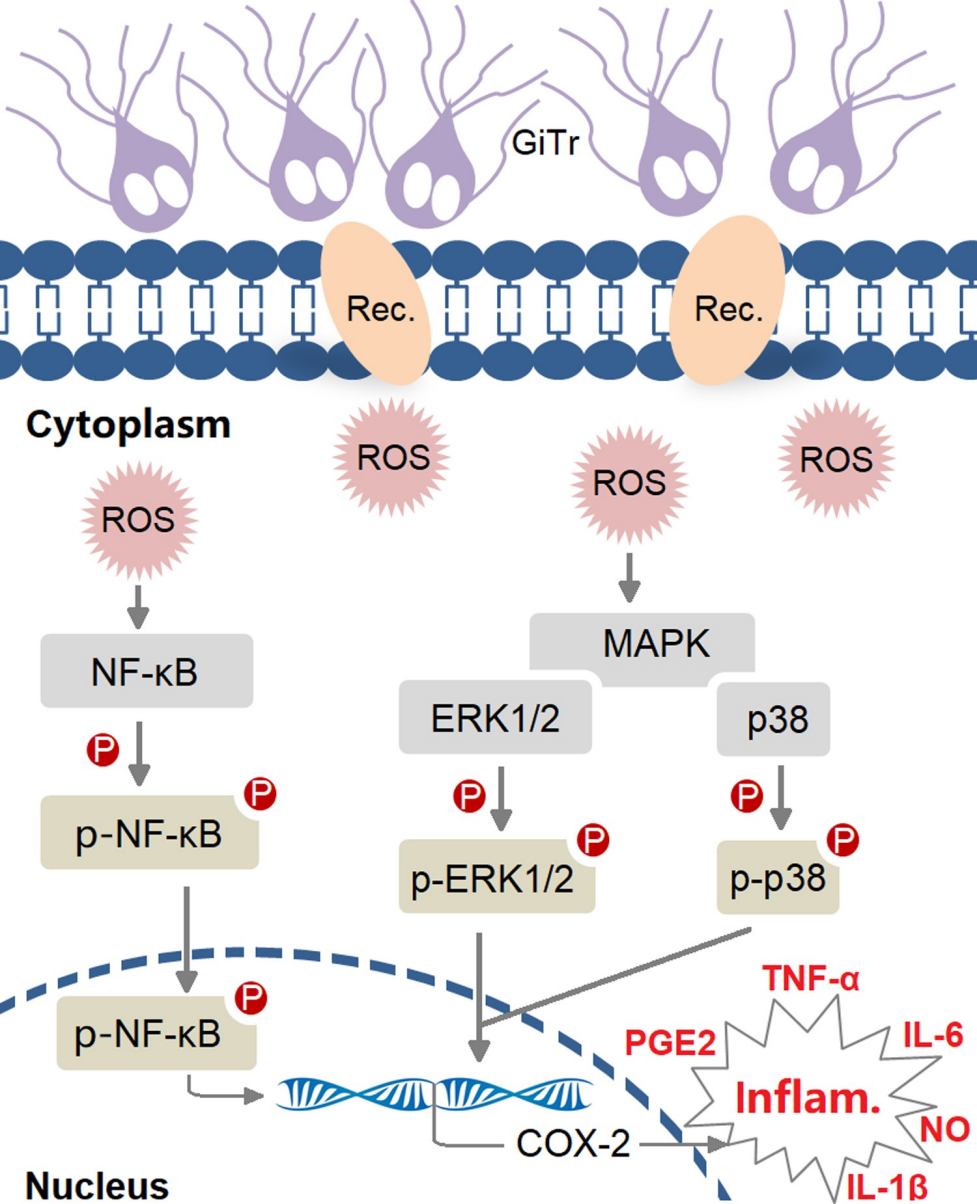
Schematic diagram illustrating critical regulators involved in *Giardia*-induced pro-inflammatory response and defense-related NO enhancement in macrophages. “GiTr” reads *Giardia* trophozoite.

In the present study, we explored if *Giardia*-induced pro-inflammatory response and NO enhancement can be modulated by COX-2, and if this process is controlled by tightly regulated signaling networks in macrophages. The results indicated that COX-2 operated as a promising modulator for promoting IL-1β, IL-6, and TNF-α expressions and PGE2 and NO production in *Giardia*-macrophage interactions. Further investigations revealed that COX-2-mediated anti-*Giardia* pro-inflammatory response and NO enhancement were under tight control of ROS-dependent MAPK/NF-κB activation (Fig 8).

Efficient immune response achieved by continuous interplay between innate and adaptive immunity is of major importance in controlling infection, and inflammation is an important and necessary part of the normal host responses to pathogens [34]. Macrophages are widely distributed innate immune cells throughout the body, which play vital roles in immunity as early effectors, initiating inflammatory response, modulating adaptive immune response, defending against microbial infections, and restoring tissue homeostasis after infection clearance [35]. Macrophages normally cooperate with neutrophils in combating microbial infections and maintaining inflammation via producing enough pro-inflammatory cytokines like TNF-α, IL-1β, IL-6, IL-12, IL-18, or IL-23 [36]. TNF-α and IL-6 expressions are elevated during *Giardia* infection, their deficiency facilitates delayed elimination of the parasite [12–15]. Here we confirmed that macrophages up-expressed IL-1β, IL-6, and TNF-α in response to *Giardia* affection. It has been noted that gut epithelial and immune cells are able to release NO that possesses antimicrobial, immunomodulatory, and cytotoxic activities [5]. NO derived from iNOS produces an inhibiting effect on *Giardia* trophozoite growth and excystation process [20], and NO derived from neuronal NOS (nNOS) induces intestinal peristalsis to promote the process of pathogen clearance [37,38]. Competitive consumption of arginine by *Giardia* declines NO release from IECs and might induce apoptosis, protecting the parasite from being eliminated [39]. Yet, in answer to *Giardia* infection, macrophages expressing arginase 1 and iNOS are recruited to the small intestine [40]. It is of interest to note in this study that, unlike IECs, macrophages triggered *in vitro* by *Giardia* showed a time-dependent increase in NO release, possibly serving as an auxiliary defense mechanism against *Giardia* infection.

During microbial infections, COX-2 is studied generally as a pro-inflammatory factor, COX-2/PGE2 has been proved to be associated with inflammasome activation and M1 polarization in *Salmonella* Typhimurium- and *Yersinia enterocolitica*-infected macrophages [41]. To the best of our knowledge, here is the first study showing *Giardia*-induced COX-2/PGE2 enhancement in macrophages. However, some studies have indicated that COX-2/PGE2 can induce anti-inflammatory profiles in cells including macrophages or tissues infected by some intracellular protozoan parasites including *Toxoplasma gondii*, *Trypanosoma cruzi*, or *Leishmania* [42–46]. The difference might be due to different modes of parasitism (extracellular versus intracellular). To date, little is known about the regulatory function and mechanism of COX-2 during microbial infections, here we demonstrated an important role of COX-2 in regulating *Giardia*-induced pro-inflammatory response and defense-related NO enhancement in macrophages.

Reflecting on our exploration, we also discovered that p38/ERK/NF-κB signaling was activated by noninvasive *Giardia* and the pathways contributed to regulation of COX-2 expression in macrophages. It has been demonstrated that invasive non-typeable *Haemophilus influenza*-induced COX-2 and PGE2 up-regulation in lung epithelial cells is correlated with p38/NF-κB activation [47]. Up-regulated COX-2 expression and PGE2 production in enterovirus type 71-infected human neuroblastoma cells are mediated via activation of MAPK/NF-κB/AP-1 signaling [48]. ROS commonly functions as a cytotoxic agent secreted from inflammatory cells to kill off invading pathogens [49]. An interesting finding of this study is that ROS accumulated in *Giardia*-exposed macrophages was biologically linked to activation of p38/ERK/NF-κB signaling and regulation of COX-2-mediated IL-1β, IL-6, TNF-α, and NO production. In reality, recombinant *Treponema pallidum* protein Tp0768 has been shown to promote generation of IL-1β, IL-6, and IL-8 by macrophages through ER stress and ROS-NF-κB pathway [50]. In addition, it was known that ROS can be activated in J774A.1 cells, peritoneal macrophages, and IECs as early as 3 h after *Giardia* exposure [16,18], and inhibition of ROS by NAC can affect macrophage pyroptotic outcome after a 12-h exposure [16] and IEC apoptotic outcome after a 6-h exposure [18]. In contrast, here we demonstrated that ROS inhibition was able to influence macrophage pro-inflammatory and defense-related responses after a 3-h or prolonged *Giardia* exposure. It is also of interest to note the function of ROS in regulating MAPK/NF-kB/COX-2 signaling as early as 3 h after *Giardia* exposure here. *Giardia*-secreted peptidyl-prolyl cis-trans isomerase B has been verified as a trigger for TLR4-dependent ROS activation and pyroptotic cell death in macrophages [16]. Likewise, continued efforts are needed to identify the potential trigger for ROS-dependent regulation observed here.

In conclusion, we reported a novel role of COX-2 in mediating *Giardia*-induced pro-inflammatory response and defense-related NO enhancement, as well as revealed its association with ROS-dependent activation of MAPK/NF-κB signaling. The findings reached in this study provide well founded insights into the complex regulatory networks of anti-*Giardia* host inflammatory responses. However, in reality, only minimal intestinal inflammation is triggered by noninvasive *Giardia* infection [5], and the exact underlying mechanism remains elusive and thus needs to be further elucidated.

## Supporting information

S1 Table Primer pairs used in qPCR analysis. (DOC)
