# Supplementary material for: COX-2 is required to mediate crosstalk of ROS-dependent activation of MAPK/NF-κB signaling with pro-inflammatory response and defense-related NO enhancement during challenge of macrophage-like cell line with Giardia duodenalis
Source: PLoS Negl Trop Dis. 2022 Apr 28;16(4):e0010402. doi: 10.1371/journal.pntd.0010402 (PMC9089906; doi:10.1371/journal.pntd.0010402)
Supplement: S1 Table — (DOC) [file pntd.0010402.s001.doc]

**Supporting information**

S1 Table. Primer pairs used in qPCR analysis.

| Gene | Accession no. | Primer (5′ to 3′) | Product size |
| --- | --- | --- | --- |
| IL-1β | NM_008361.4 | F: GCTGCTTCCAAACCTTTGAC  R: AGCTTCTCCACAGCCACAAT | 121 bp |
| IL-6 | NM_001314054.1 | F: CACATGTTCTCTGGGAAATCG  R: TTGTATCTCTGGAAGTTTCAGATTGTT | 116 bp |
| TNF-α | NM_001278601.1 | F: CAGGCGGTGCCTATGTCTC  R: CGATCACCCCGAAGTTCAGTAG | 89 bp |
| COX-2 | NM_011198.4 | F: GTGCCTGGTCTGATGATGTATG  R: TGAGTCTGCTGGTTTGGAATAG | 121 bp |
| iNOS | NM_010927.4 | F: ACATCGACCCGTCCACAGTAT  R: CAGAGGGGTAGGCTTGTCTC | 177 bp |
| β-actin | NM_007393.5 | F: AGTGTGACGTTGACATCCG  R: GCAGCTCAGTAACAGTCCGC | 298 bp |
